# A Handheld Visible Resonance Raman Analyzer Used in Intraoperative Detection of Human Glioma

**DOI:** 10.3390/cancers15061752

**Published:** 2023-03-14

**Authors:** Liang Zhang, Yan Zhou, Binlin Wu, Shengjia Zhang, Ke Zhu, Cheng-Hui Liu, Xinguang Yu, Robert R. Alfano

**Affiliations:** 1 Department of Neurosurgery, Medical School of Nankai University, Tianjin 300071, China; 2 Department of Neurosurgery, PLA General Hospital, Beijing 100853, China; 3 Department of Neurosurgery, Air Force Medical Center, Beijing 100142, China; 4 Physics Department and CSCU Center for Nanotechnology, Southern Connecticut State University, New Haven, CT 06515, USA; 5 JRME Co., Ltd., Taizhou 225300, China; 6 Institute of Physics, Chinese Academy of Sciences (CAS), Beijing 100190, China; 7 Institute for Ultrafast Spectroscopy and Lasers, Department of Physics, The City College of the City University of New York, New York, NY 10031, USA

**Keywords:** portable optical-fiber-probe Raman analyzer, human brain, glioma, visible resonance Raman (VRR), intraoperative, optical biopsy, optical molecular pathology

## Abstract

**Simple Summary:**

Real-time diagnosis tools and methods are desired to aid in the intraoperative grading of glioma and tumor boundary identification to achieve safe maximal tumor removal. Raman spectroscopy is an optical method for real-time glioma detection, but few studies use fresh glioma tissue for biochemical analysis. This study is the first investigation of human glioma using a portable VRR-LRR^TM^ Raman analyzer under quasi-clinical conditions, and reveals significant spectral differences between normal (control) and different grades of glioma. A principal component analysis–support vector machine (PCA-SVM) machine learning method was used to distinguish glioma tissues from normal tissues and different glioma grades. The accuracy in identifying glioma from normal tissue was over 80%, compared with histopathology as the gold standard. This result validates the possibility of glioma diagnosis using fresh tissue and provides instant feedback for neurosurgeons in guiding maximal safe resection, and it may support the translation of this portable tool for in vivo and real-time use in tissue biochemical analysis.

**Abstract:**

There is still a lack of reliable intraoperative tools for glioma diagnosis and to guide the maximal safe resection of glioma. We report continuing work on the optical biopsy method to detect glioma grades and assess glioma boundaries intraoperatively using the VRR-LRR^TM^ Raman analyzer, which is based on the visible resonance Raman spectroscopy (VRR) technique. A total of 2220 VRR spectra were collected during surgeries from 63 unprocessed fresh glioma tissues using the VRR-LRR^TM^ Raman analyzer. After the VRR spectral analysis, we found differences in the native molecules in the fingerprint region and in the high-wavenumber region, and differences between normal (control) and different grades of glioma tissues. A principal component analysis–support vector machine (PCA-SVM) machine learning method was used to distinguish glioma tissues from normal tissues and different glioma grades. The accuracy in identifying glioma from normal tissue was over 80%, compared with the gold standard of histopathology reports of glioma. The VRR-LRR^TM^ Raman analyzer may be a new label-free, real-time optical molecular pathology tool aiding in the intraoperative detection of glioma and identification of tumor boundaries, thus helping to guide maximal safe glioma removal and adjacent healthy tissue preservation.

## 1. Introduction

Glioma is the most common brain neoplasm and represents around 27% of all intracranial tumors [1,2]. Most gliomas are high-grade and malignant [3,4] and feature an infiltrative growing pattern and aggressive behavior with a dismal prognosis. Despite the advancement of surgical resection and adjuvant radio-chemotherapy, the survival outcome has not been improved and is far from desirable. The median overall survival (OS) is only 3 years and 12–18 months for grade 3 and 4 glioma, respectively, and the 5-year survival rate is only 6.8% for glioblastoma (GBM) [1,5,6,7].

The current brain tumor treatment starts with surgical resection, which is the most critical first step in the comprehensive treatment of glioma. Maximal safe surgical resection plays a key role in the management of patients with glioma. Increasing evidence demonstrates that a greater extent of resection has a positive correlation with the patient’s progression-free survival (PFS) [7,8,9,10]. However, the infiltrative growing nature of glioma makes it difficult to distinguish normal tissue from the tumor region, thus preventing neurosurgeons from achieving total resection [10].

The most commonly used auxiliary techniques during surgery, such as stereotaxis [11], can not only provide preoperative biopsies for pathological analysis but also help the surgeon to better identify and calibrate the location of the tumor, which has a great advantage compared with traditional microsurgery. However, due to the limitations of positioning, the biopsy specimens obtained in a stereotactic surgery are small and do not represent the overall nature of the tumor, leading to an underestimation of high-grade brain tumor malignancy. Another auxiliary technique is intraoperative ultrasound technology, which was first applied in 1980, attracting the attention of neurosurgeons [12,13]. Intraoperative ultrasound has many advantages, such as clear images for surgeons; it is simple, flexible, low-cost, and allows intraoperative real-time positioning, and the procedure helps neurosurgeons to remove the lesions. However, even using intraoperative ultrasound, it is still difficult to determine the real boundary of a glioma. The invasive growth of tumor cells often occurs in the aggressive area of the tumor.

Currently, a definitive diagnosis is usually made by histopathological and molecular examination retrospectively, but it is very time-consuming. Real-time diagnosis tools and methods are needed to aid in the intraoperative grading of glioma and tumor boundary identification, since an essential balance between maximal tumor removal and the risk of disability is needed intraoperatively. Such techniques will allow the neurosurgeon to tailor their surgical strategy according to each patient’s profile [7,10,14].

Among the newly explored molecular diagnosis methods, Raman spectroscopy can analyze the changes in the chemical compositions of different lesions in human tissue and the small changes in protein structure and nucleic acids [15,16,17,18,19,20] using intrinsic molecular fingerprints in situ, being label-free and near-real-time [17,21,22,23,24,25,26,27,28,29,30,31,32,33,34], and at a low cost. Raman spectral changes can reveal the metabolic processes of brain tissue [24,25,30] and can potentially be used for margin assessment, even during surgery [24,30,31,32,33,34,35]. Raman spectroscopy techniques have been used to study human bladder cancer, esophageal cancer, gastrointestinal cancer, cervical cancer, skin melanoma lesions, lung cancer, breast cancer, and brain tumors, not only ex vivo but also in vivo [15,16,19,20,21,22,22,23,26,27,28,31,33,36,37,38,39,40]. Besides solid tumors, Raman spectroscopy has also been used to detect vulnerable atherosclerotic plaques, atherosclerotic abdominal aortic tissues, and nerve tissues, and even the cerebrospinal fluid and serum of patients [41,42,43,44].

We have developed a novel optical spectroscopic technique based on visible resonance Raman (VRR) spectroscopy that can probe the specific molecular vibration bonds in a tissue and provide information to make an optical pathology diagnosis. This new optical biopsy technique using 532 nm wavelength excitation has been used for the investigation and diagnosis of human lesions since 2011 and has shown unique advantages [26,34,40,41,45,46,47]. Besides the existing advantages of conventional spontaneous Raman (SR), near-infrared Raman (NIR), and ultraviolet resonance Raman (UVRR) methods, VRR has additional advantages over other Raman techniques [48]. This is due to the use of 532 nm light being in-resonance or near-resonance with the native bio-molecular absorption bands in biological tissue and cells, which can enhance the Raman signals from biomolecules by 10 to 1000 times. VRR can be used to discover and study changes in the composition of biomolecules locally and reveal their variations in the concentration and spatial distribution based on the spectral fingerprints of the biochemical vibrational bands. In recent years, some research groups have begun to use 532 nm as a light source for Raman for basic research in biomedicine, such as surface-enhanced Raman spectroscopy (SERS), using targeted label methods [49,50,51]. Based on our previous studies, we have developed a new portable VRR-LRR^TM^ Raman analyzer (LRR2000) with a handheld optical-fiber probe that shows comparable diagnostic ability for glioma in mice in vivo and for human brains ex vivo [47,52,53]. In this manuscript, we report the evaluation of the VRR-LRR^TM^ analyzer in identifying human glioma grades and margins under quasi-clinical conditions.

There are similar systems that have been developed based on Raman techniques and are promising for intraoperative cancer diagnosis, such as the landmark handheld Raman spectroscopy probe developed by Desroches et al. for intraoperative glioma detection [36], an in vivo pathology system based on confocal laser scanning microscopy–Zeiss CONVIVO [37] and Stimulated Raman Histology (SRH) [38,54]. Desroches’ system uses an NIR 785 nm wavelength with a power of 37–64 mW for excitation. Compared to this system, we believe that our VRR-LRR^TM^ system can improve the signal to noise ratio (SNR) due to the resonance effect. The Zeiss CONVIVO can be used for in vivo pathology. The system collects a fluorescence signal from the tissue with a contrast agent, fluorescein sodium. It provides real-time images of the tissue microstructure, which can then be evaluated by trained pathology experts. Compared to CONVIVO, our VRR system is label-free and does not use any contrast agent. It provides chemical changes at the molecular level. The molecular information can then be used for classification using machine learning methods. SRH is an important and promising Raman imaging technique based on stimulated Raman scattering (SRS) for ex vivo diagnosis. SRS typically uses a high-cost pico- or femto-second ultrafast pulsed laser as the light source. Images are collected at selected wavenumbers, such as 2845 cm^−1^ and 2930 cm^−1^ [38], to form images due to macromolecules such as lipids, proteins, and DNA, etc. On the contrary, the VRR system is low-cost and can complete the full scan of the Raman spectra, including the fingerprint and high-wavenumber regions, with a single quick scan.

## 2. Materials and Methods

In this study, the raw experimental data were collected from patients who underwent surgical resection of brain glioma from October 2019 to February 2020. The study was approved by the Institutional Review Board (IRB) of the PLA General Hospital. Consent was obtained from all patients. A total of 63 fresh unprocessed tissue samples (considered quasi-clinical condition) were collected from 52 adult patients (grade 1: 1 patient; grade 2: 7 patients, grade 3: 17 patients; grade 4: 27 patients) with an age range of 18–78. The samples included 4 additional glioma tissues collected from grade 4 patients, and 7 normal control tissues collected from negative margins of gliomas. The sub-class breakdown of the glioma patients is as follows: grade 1, including 1 pilocytic astrocytoma; grade 2, including 2 astrocytomas IDH^mutant^ (isocitrate dehydrogenase, IDH) and 5 oligodendrogliomas IDH^mutant^; grade 3, including 4 astrocytomas IDH^mutant^ and 13 oligodendrogliomas IDH^mutant^; and grade 4, including 26 GBMs IDH^wild-type^ and astrocytomas IDH^mutant^ and 1 diffuse midline glioma. Of these patients, 32 were male (61.5%) and 20 female (38.5%), with an age of 48.4 ± 14.1 years. The classification of these tumors was made according to the 2021 WHO classification [4]. Our study was conducted before the 2021 WHO classification was published. The major changes from the 2016 to 2021 classification included the restriction of the diagnosis of GBM only to tumors that are IDH^wild-type^ and the reclassification of tumors previously diagnosed as GBMs IDH^mutant^ now as astrocytomas IDH^mutant^, grade 4 [55,56]. Therefore, according to the 2021 classification, the grade 4 tumors in this study included GBM and IDH-mutant astrocytoma based on the molecular testing results of the tissue samples.

A total of 2220 raw VRR spectra (grade 1: 15 spectra, grade 2: 359 spectra, grade 3: 717 spectra, grade 4: 1103 spectra, and normal control: 26 spectra) were acquired from the samples using LRR2000 with different acquisition parameters for exposure and averaging.

**Figure 1:**
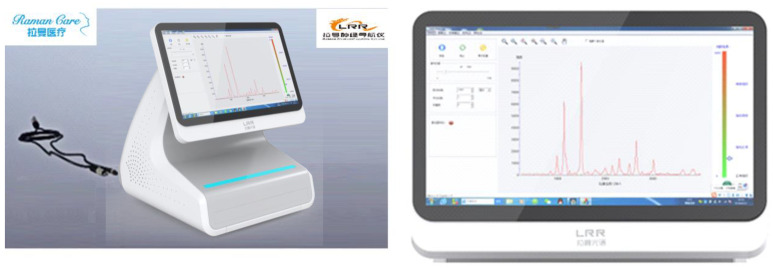
Photographs of the portable VRR-LRR^TM^ analyzer with a label-free optical-fiber probe along with example experimental data. The analyzer is 32 cm × 33 cm × 38 cm in size and weighs 7.5 kg, with a standard cable of probe of 2 m. The screen displays recorded signals and test results, along with flickering color and ringtones in real time (right side of screen). When the surgical resection is challenging, the tissue may be located at the border of the tumor with the real-time display of classification; the VRR-LRR^TM^ handheld probe provides the results and helps the surgeon to decide whether to remove the tissue [45].

**Figure 2:**
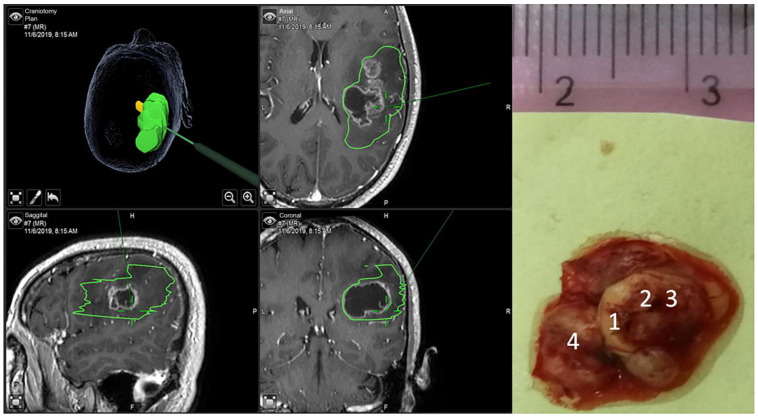
Magnetic resonance imaging (MRI) data were transferred to the neuro-navigation system (Brainlab, Germany), and demonstrated a large glioma in the left parietal lobe. The sample was obtained under a microscope with the guidance of intraoperative neuro-navigation. The spectra were obtained from 4 sites in the fresh unprocessed specimen immediately after resection. Spectra acquisition was performed using the VRR-LRR^TM^ analyzer. The exposure time was 3 s, excitation laser wavelength 532 nm, with full scan range from 200 to 4000 cm^−1^ for analysis of VRR spectra. The tumor was pathologically verified as glioblastoma (WHO grade 4).

Samples were obtained under a microscope with the guidance of neuro-navigation (Brainlab, Munich, Germany) and were transferred immediately to the adjacent theater for spectral measurements using the VRR-LRR^TM^ (LRR2000) Raman analyzer, as shown in Figure 1. This portable Raman analyzer uses a 532 nm excitation wavelength. An optical-fiber probe was used to focus the laser beam to a 200 μm focal spot with laser power of 5 mW on the sample surface. The same probe was used to collect the scattered signals from the sample surface. The VRR spectra of a specimen were measured from multiple sites (3 or more) according to the sample size, with an acquisition time of 1 s, 3 s, or 5 s, and a spectral resolution of 8 cm^−1^ in the range of 200 to 4000 cm^−1^, as shown in Figure 2.

**Figure 3:**
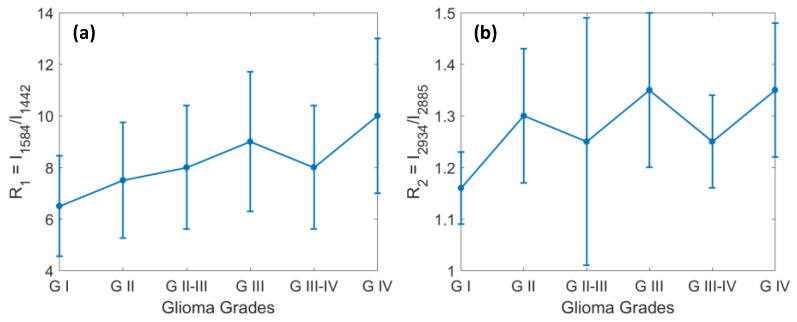
Average VRR spectral data plots: the ratios of (**a**) R1 = I_1584_/I_1442_ and (**b**) R2 = I_2934_/I_2885_ from glioma tissues with increasing malignancy. G I, glioma grade 1; G II, glioma grade 2; G II-III, glioma grade 2–3; G III, glioma grade 3; G III-IV, glioma grade 3–4; and G IV, glioma grade 4.

In the left two columns of Figure 2, the top right, bottom left, and bottom right images are selected axial, sagittal, and coronal planes of MRI images of the tumor. The boundary of the tumor is outlined. The image in the top left is a reconstructed 3D image of the tumor. The third column of Figure 3 shows a picture of the tumor tissue along with the image of a ruler, where the numbers 1–4 indicate four spectral acquisition sites. The data evaluation and processing used LRR2000^TM^-1, JRME, the Origin 2015 software, and MATLAB.

All grades of glioma samples were included in the peak analysis. For classification using machine learning and statistical analysis, grade 1 glioma samples were omitted due to the small sample size, one grade 2 patient was omitted due to uncertainty in histopathology, and only the spectra collected using a 5-s exposure time and average of 3 acquisitions were used. More details of the samples used for the classification will be provided in a later section.

After the spectral measurements using LRR2000, all samples were snap-frozen and restored under −80 °C conditions. Then, a Horiba LabRAM HR-Evolution micro-confocal Raman system (HORIBA, Longjumeau, France) was used to collect Raman data from some of the samples for comparison. The HR-Evolution Raman system also uses a 532-nm excitation wavelength and a laser power of 1.0 mW and a 50× objective. The spot size of the beam on the sample surface was ~2 μm. The spectral resolution was ~2 cm^−1^. A single scan was carried out in the process of spectral collection with an integration time of 5 s. A section of an adjacent specimen was collected and stained with hematoxylin and eosin (H&E) and underwent molecular examination by a trained neuropathologist. The histopathological diagnosis was in accordance with the 2021 WHO classification as the gold standard.

## 3. Glioma Grading Using Machine Learning

To perform glioma grade classification, VRR spectra collected with a 5-s integration time were used. Classification models based on individual peaks and whole spectral profiles were both performed and compared. For classification based on individual peaks, a selective set of peak intensities was used as features. For classification based on whole profiles, the spectra were first analyzed using principal component analysis (PCA), and then a selective set of principal component (PC) scores was used as the features to build models [45]. Various combinations of the features were compared to select the optimal combination of the features. The method to select the model with the optimal feature combination is described below. Support vector machines (SVMs) with Gaussian kernels were used to train the classifiers [45]. SVM attempts to find a hyperplane to separate two classes with the largest distance from the nearest class members (data points). When SVM is used for multi-class classification, a model is trained with a hyperplane to separate each class from all other classes. A composite hyperplane to separate multiple classes can be created and visualized by combining all the individual hyperplanes. When a data point is tested, it will be evaluated using all the individual models. The model that results in the highest score will be used to determine the predicted class.

To reduce sample selection bias, leave-one-out cross-validation (LOOCV) [45,57] was used to evaluate the classification performance. To perform LOOCV, each time, one individual data point corresponding to a spectrum was removed from the dataset. The rest of the dataset was used to train the SVM model. The removed data point was then classified using the model for testing. This process was repeated for all data points. In the end, statistical measures including sensitivity, specificity, and accuracy, along with area under receiver operating characteristic (ROC) curve, i.e., AUROC, were calculated based on the results of all tests as an overall evaluation of the classification performance [45,57]. Since there are many features (Raman peaks or PCs) available, we evaluated different combinations of features to find the optimal combination by comparing the classification result with LOOCV. All of the computations for peak-SVM and PCA-SVM were performed in MATLAB R2017b using the built-in machine learning toolbox.

## 4. Analysis and Results

VRR spectra of glioma with grade 1 to grade 4 from human brain tissues were measured. To perform preliminary peak analysis, all RR spectra were first normalized by the peak at 1001 cm^−1^ of phenylalanine, which has a relatively stable intensity and position in different environments [45]. The spectral data collected by LRR2000 were qualitatively compared with those collected using the Horiba LabRAM system to validate the Raman peaks. The data collected using the Horiba system are not shown in this manuscript. The changes in proteins and lipids indicated by the average values of the two peak ratios R1 = I_1584_/I_1442_ and R2 = I_2934_/I_2885_ were analyzed. An enhancement was observed due to the Fermi resonance doublet of amide B and amide A under the quasi-clinical conditions. One of the new biomarkers at the RR mode of 3058 cm^−1^ in the high-wavenumber region, which arises from tryptophan, was also analyzed.

### 4.1. The Changes in Lipids and Proteins for Identification of Grades 1 through 4 of Glioma 

The changes in proteins and lipids indicated by the average values of the two peak ratios R1 = I_1584_/I_1442_ and R2 = I_2934_/I_2885_ during the evolution of the metabolism of gliomas are shown in Figure 3. The standard deviations of the peak ratios are within 30% and 15% for R1 and R2, respectively. The ratio R1 for G0 is less than 1, and R2 is ~1.1, which is close to that of GI. For better visualization, we have omitted G0 from the plots. The two Raman peak ratios in Figure 3 indicate the ratio of proteins to lipids vs. glioma grades that were calculated from the VRR spectra data. Figure 3a shows the VRR spectral peak intensity ratio of R_1_ = I_1584_ to I_1442_ versus glioma grades. The graph shows a positive correlation between the peak intensity ratio and the glioma grade. For instance, from the ratio values, we can see that the strength of the saturated lipid bond, which is related to the hydrophobic chains of lipids near 1442 cm^−1^, becomes relatively weaker with higher grades [58,59], while the other VRR molecular fingerprint of 1584 cm^−1^, which is contributed from proteins of tryptophan, mitochondria, hemeproteins, and nucleic acids, shows a resonance enhancement and a tendency to increase with the grade [34,45].

Figure 3b shows the VRR spectral peak intensity ratio of R_2_ = I_2934_ to I_2885_ versus glioma grades. The Raman modes of 2934 and 2885 cm^−1^ are symmetric stretches that correspond to methyl (-CH3) and methylene (-CH2-), respectively [45]. Comparing grade 1 through grade 4, an increase in the intensity ratio was observed and it gradually increased in gliomas with increasing grades. This indicates that the relative peak intensity of 2885 cm^−1^ was reduced and the peak at 2934 cm^−1^ became stronger compared to that at 2885 cm^−1^.

These changes can be explained by the fact that the composition and conformation of saturated lipids and proteins (including amino acids and lipoproteins) in cells and tissues change during the evolution of the metabolism of glioma from grade 1 to grade 4 [45]. It should also be noted that there is a significant fluctuation, especially from grade 2 through grade 4 in R_2_. The fluctuation may be attributed to the fluctuation in the chemical composition of each grade of glioma, the condition of the samples in the intraoperative study, and the relatively lower signal to noise ratio of the home-built system compared to the commercial confocal systems. These ratios should be further verified in future studies with large datasets. In summary, the above two ratios versus glioma grades are consistent with our previous report of the criteria for glioma grading by the VRR spectroscopy technique, except that no significant decrease in the ratio value was observed in the glioma grade 4 data in this study [34,45]. The ratios R_1_ and R_2_ used as diagnostic criteria reveal the relative concentration changes between proteins and lipids during the metabolic processes of glioma. The result clearly indicates that the concentration of saturated lipids was reduced in the tumors. This method and the results are consistent with reports in the literature [51].

### 4.2. Identification of Glioma Margin by Carotenoids and the Ratio of Protein to Lipids

The resonance-enhanced intrinsic molecular fingerprints with intense RR modes at 1159 cm^−1^ (ν_s_ C-C) and 1517 cm^−1^ (ν_s_ C=C) mainly arise from lutein in the carotenoids of the human brain. Carotenoids play an important role in the antioxidant defense system in the healthy brain [60]. Carotenoids have been extensively studied and confirmed as a cancer biomarker in human organs using Raman, especially resonance Raman spectroscopy, to identify cancers of the skin, rectum/colon, lung, breast, and brain [42,61,62,63]. Johnson et al. reported the important role of carotenoids, and particularly the presence of lutein and zeaxanthin, in normal brain tissue [64,65,66].

**Figure 4:**
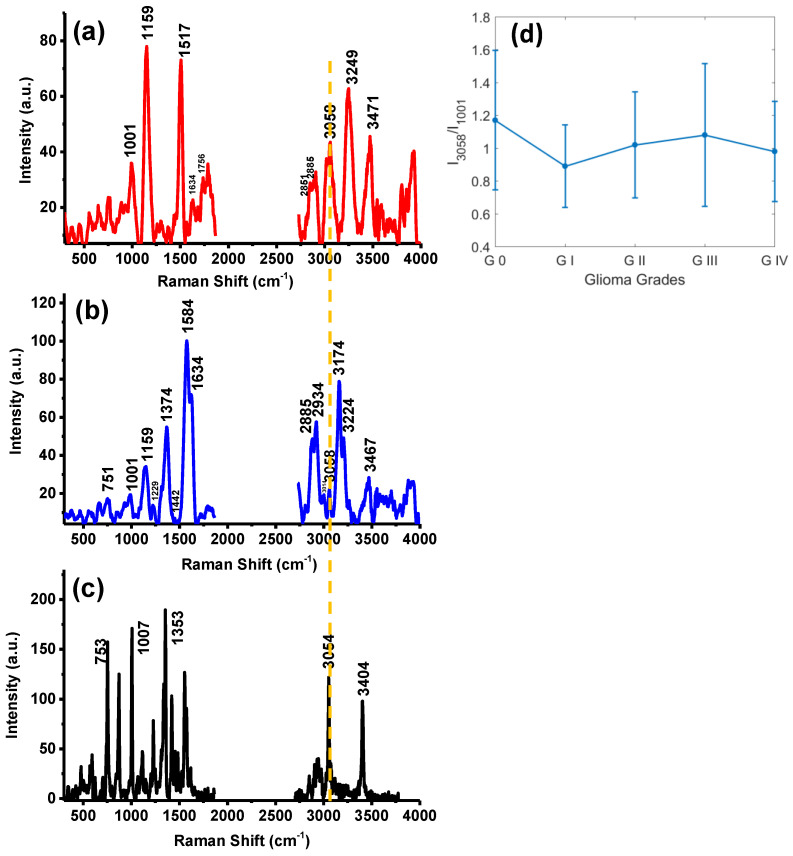
Typical VRR spectra of (**a**) normal/control of glioma margin tissue, (**b**) glioma grade 4 tissues, and (**c**) tryptophan powder, along with (**d**) the content of the RR mode around 3058 cm^−1^ of tryptophan versus glioma grade. Raman peak of 3058 cm^−1^ was normalized by RR peak at 1001 cm^−1^ of phenylalanine. G0, normal; G I, grade 1; G II, grade 2; G III, grade 3; and G IV, grade 4.

In this study, the normal/control VRR spectra were collected from the margins of different grades of glioma. A typical VRR spectrum of the control site is shown in Figure 4a. From Figure 4a, the relatively sharp and enhanced VRR peaks of 1159 and 1517 cm^−1^ arising from carotenoids can be observed at the cancer–normal tissue interface of the specimens. Rich fatty acid peaks at 2851 and 2885 cm^−1^ are stronger than the protein bands at 1001, 1634, and 2934 cm^−1^ in intensity. In a comparative study, Verdiyan et al. collected Raman spectra from the myelin sheath of sciatic nerve fiber tissue using 532 nm laser excitation and observed peaks at 1160, 1520, 2850, 2885, and 2935 cm^−1^, respectively [42]. Figure 4b is the typical VRR spectrum from glioma grade 4 tissue. Figure 4b shows that the feature peaks at 1159 and 1517 cm^−1^ of carotenoids both underwent remarkable decreases, with the 1517 cm^−1^ peak almost disappeared. By comparing Figure 4a,b, a clear decrease is observed in the intensities of the Raman peaks with the increase in the glioma grade. This indicates the progression of the mutation process in the glioma and can be used as a biomarker to identify the glioma margin. Our observation shows that in order to identify the boundary of a tumor, one can combine the resonance-enhanced characteristic Raman resonance modes 1159 and 1517 cm^−1^ of carotenoids, and the ratios of proteins to lipids. The boundary values of the ratios are R_1_ less than 6.0 and R_2_ less than or equal to 1.0. These results agree with our previous findings obtained with the LRR2000 and HR800 Raman systems ex vivo [34,45,52,53].

### 4.3. The New VRR Biomarkers of Glioma in the High-Wavenumber Region

Two new biomarkers were found in gliomas that were strongly enhanced RR modes at 3174 cm^−1^ and 3224 cm^−1^, associated with natural molecular vibrational bands assigned to the N-H stretching vibration of the amide B protein and the NH_2_/O-H symmetric stretching vibration of the amide A protein/amino acid glutamine, respectively, as shown in Figure 4b. In this study, we propose that the amide A band at 3224 cm^−1^ is assigned to a Fermi resonance doublet (R-doublet), and its second component is assigned to amide B at 3174 cm^−1^ [67].

We also observed that the intensity changes and frequency shifts of the two vibrational modes at 3174 and 3224 cm^−1^ were approximately proportional to the VRR vibration modes of 1584 and 1634 cm^−1^ (amide I). This phenomenon could be caused by two possible sources. First, the enhancement of the peaks is a result of Fermi resonance. We propose that, it is due to anharmonicity that two relatively strong bands at around 3174 and 3224 cm^−1^ were observed, although only one strong band at approximately 3174 cm^−1^ (overtone) for the fundamental vibrational band near 1584 cm^−1^ was expected. Second, under the quasi-clinical test conditions, the surface of the tissue was covered by water, which may work as a solvent to produce a solvent effect. Due to the external molecular field, the interaction of the hydrogen-bonded NH groups with the amide B band may affect the Fermi resonance and cause the frequency shift and intensity change of the Fermi resonance doublet [68]. This phenomenon is revealed in Figure 4a,b. Comparing Figure 4a,b, one finding is that, in Figure 4b, in the high-grade glioma tissue, the resonance-enhanced vibration modes of 1584 and 1634 cm^−1^ correspond to the Fermi resonance-enhanced doublet modes of 3174 and 3224 cm^−1^. However, in Figure 4a, in the normal control VRR spectrum, the weakened 1584 and 1634 cm^−1^ peaks correspond to the almost disappearing 3174 cm^−1^ peak and the 3249 cm^−1^ peak, which shifted to a higher-wavenumber position compared to grade 4. This is an interesting result and will be further verified.

Figure 4 shows a new, important biomarker for gliomas, which is a resonance Raman mode of tryptophan at 3058 cm^−1^. In the literature, it has been shown that the heterocyclic amino acid tryptophan in human brain gliomas is an essential factor and plays a critical role in the metabolic process [69,70]. Tryptophan metabolism involves the kynurenine pathway [71,72,73,74]. The catabolism of >95% of tryptophan in the brain metabolic process takes place through the kynurenine pathway. Previous studies have found that the degradation of tryptophan and the tryptophan metabolite, kynurenine (Kyn), can inhibit the antitumor immune response. The enzyme of tryptophan-2,3-plusoxidase can act directly on glioma cells and promotes tumorigenesis [73,75]. The change in the 3058 cm^−1^ peak in Figure 4 reflects the change in the concentration of tryptophan during its catabolism in glioma of different grades.

Figure 4a–c include the typical VRR spectra from normal/control samples of glioma margin in (a); a VRR spectrum of glioma grade 4 in (b); and a VRR spectrum of tryptophan powder in (c). The dotted line inserted in the spectra of Figure 4a–c marks the position of the RR mode of 3058 cm^−1^ in the spectrum of normal brain tissue, glioma grade 4, and tryptophan. Figure 4d shows the two metabolic stages of tryptophan. We observed that, first, in normal/control brain tissue, the concentration of tryptophan is greater than that in gliomas. This decrease in tryptophan may be due to tryptophan depletion in cancers [76]. However, the decrease was not observed in our ex vivo study [45]. It may be due to the difference in the microenvironment between ex vivo frozen tissue and intraoperative fresh tissue. This will be verified in further studies. Second, in glioma tissue, the concentration of tryptophan increases slightly with the malignancy of tumors. Many studies have shown that the degradation of tryptophan is a mechanism that tumors select to achieve immune escape [75,77]. There are more large amino acid transporters on the cell membranes of more aggressive cancer cells; therefore, tryptophan can be taken up more efficiently in such cells than others from the surrounding environment. The RR mode of 3058 cm^−1^ reveals that tryptophan in glioma tissues accumulates during tumor progression from GI to GIV. However, the concentration of tryptophan in GIV decreases rapidly. This may be due to the metabolism of high-grade tumors, with the changes in cell apoptosis and tissue necrosis, where the concentration of tryptophan is also reduced. A similar phenomenon was also observed in ex vivo studies [45].

According to Figure 4c and the literature [78], the Raman bands at 873 cm^−1^ (not labeled in Figure 4c), 3058 cm^−1^, and 3404 cm^−1^ are the contributions of tryptophan. Figure 4a is the VRR spectrum of normal brain tissue. However, the 873 and 3404 cm^−1^ bands are significantly weaker or even disappeared in Figure 4a, which is different from the 3058 cm^−1^ band. We propose this is due to the environment in tissue being different from that of pure tryptophan chemical. The 873 cm^−1^ band is an indole ring vibration mode associated with N_1_H bonds and sensitive to hydrogen bonding. The 3404 cm^−1^ band is assigned to the indole-stretching vibration mode ν(NH), and is also sensitive to hydrogen bonding. We propose that under 532 nm excitation, both the 873 and 3404 cm^−1^ bands are in strongly hydrogen-bonded states, which leads to significant weakening in their Raman modes. This will be further verified in future studies.

The most intense Raman band of water is also at approximately 3400 cm^−1^ [79], which is close to one of the high-wavenumber tryptophan peaks. The contribution of water, especially in the high-wavenumber region, needs to be further discussed.

### 4.4. PCA-SVM and Peak-SVM Analyses

Although the proposed biomarkers that we observed above are promising for the diagnosis of glioma, a more robust method to perform the diagnosis would be to evaluate all present Raman peaks and the whole profiles of the spectra. We present an analysis based on individual Raman peaks and whole profiles, and compare the performance of classifications using various combinations of spectral features based on peaks and whole profiles for a thorough evaluation.

To perform classification using machine learning, the spectra collected using a 5-s exposure time and three-acquisition average were used. The original samples include grey matter and white matter in normal brain tissues, and glioma tissues at grades 1, 2, 3, and 4. Since there was only one case of glioma grade 1, it was omitted from the classification. Normal tissue samples were white matter tissues that were near gliomas. Due to histopathology conventions, grade “2–3” is treated as grade 3, and grade “3–4” as grade 4. In summary, the data used for classification included normal tissue and glioma grades 2, 3, and 4. In total, there were 359 spectra from 59 tissue samples selected from the 52 patients. The number of samples and number of spectra are summarized in Table 1 below.

The data were first baseline-subtracted and normalized to the root-mean-squares (RMS) of each spectrum for pre-processing. The baseline subtraction was performed using a novel algorithm, the Sensitive Nonlinear Iterative Peak (SNIP) clipping algorithm [80,81]. Peak intensities were obtained from the pre-processed data. PCA was then performed, with PC scores obtained. Both the intensities of selected peaks and PC scores from PCA were used as the features for classification in peak-SVM and PCA-SVM, respectively. To perform PCA-SVM, the pre-processed data were analyzed using PCA. The optimal combinations of the features, either peaks or PC scores, were found by evaluating the models based on different combinations of the features with LOOCV for comparison [57]. The optimal combinations of peaks and PCs were then used in another LOOCV to evaluate the performance of classification. In the peak combinations, 1512 cm^−1^ is considered to be due to carotenoids since the resolution of the system is 8 cm^−1^. Raman modes at 1302 cm^−1^ can be assigned to both lipids and proteins; 1371 cm^−1^ can be assigned to saccharide, DNA/RNA, and lipids; 1620 cm^−1^ can be assigned to proteins; 3002 cm^−1^ can be assigned to lipids. The significance of the peaks selected in the optimal combination will be further evaluated in future studies.

**Figure 5:**
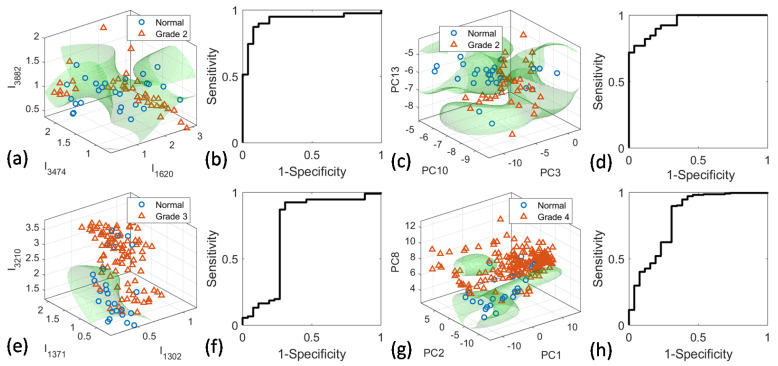
Binary classification of normal tissue and individual tumor grades using optimal combinations of peaks or PCs: (**a**,**c**) normal vs. grade 2 using peaks and PCs, respectively; (**e**) normal vs. grade 3 using peaks; (**g**) normal vs. grade 4 using PCs; (**b**,**d**,**f**,**h**) are the LOOCV ROC curves corresponding to (**a**,**c**,**e**,**g**), respectively.

Binary classification was performed to distinguish all tumor grades from normal tissue, as well as each individual tumor grade from normal tissue. Multi-class classification was also performed to classify all four types of samples, and the three tissue types with normal tissues excluded. The classification results, including sensitivity, specificity, and accuracy, as well as AUROC with LOOCV, are summarized in Table 1 for both peak-SVM and PCA-SVM. The scatterplot with the SVM classifier hyperplane is shown in Figure 5 for classification between normal and grades 2, 3, and 4, respectively.

The results in Table 2 show that the performance of peak-SVM and that of PCA-SVM is similar, without significant differences in most cases. The overall accuracy is over 80% for all binary classifications. The discrimination between normal and grade 2 glioma tissues was found to be better than other binary classification results. This may be due to the increasing heterogeneity of the tumor tissues, which caused a larger spread for the data points of higher grades. The sensitivity of the classification is over 90% for all binary classifications. However, the specificity is as low as 50% for normal vs. cancer (including all grades), with the highest for normal vs. grade 2 at ~80%. For multiclass classifications, the accuracy for grade 4 spectra is over 70% for peak-SVM and over 90% for PCA-SVM. However, the accuracy for other tissue types is low, at approximately 50% at the highest. This means that simultaneously distinguishing all tissue types is challenging. This is because there is significant overlapping of data among different tissue types.

## 5. Discussion

In freshly tested human brain tissue, we found Raman modes as evidence of water and hemoglobin. This is an important problem that needs further study for intraoperative use of VRR or in vivo use in the future. The water concentration is greater in gliomas than grey and white matter. This concentration is different in different tissues. These changes have diagnostic importance. For example, glioma often causes peritumoral edema and the adjacent normal brain tissue is often swollen. Moreover, when additional water is used to flush the operating field, the bipolar nature of water molecules can cause the surface of a tissue to carry more water, which may be shown in the high-wavenumber Raman biomarker test [67]. Another important phenomenon is that the Raman signal from hemoglobin is usually resonance-enhanced with 532 nm excitation. High-grade gliomas usually need more feeding arteries for their rapid growth with abundant de novo blood microvessels. Blood or hemoglobin may have diagnostic value for the malignancy of the glioma tumor margin. We should bear in mind this confounding factor and identify the tissue signal with the contribution of blood. Water and blood are two significant components in the lesion’s metabolism that are worthy of further study in clinical Raman testing in vivo.

In our previous research, it was shown that there were changes in the intensities of the RR peaks of carotenoids (1159 and 1517 cm^−1^) in the glioma boundary. β-carotene plays an important role in the antioxidant defense system in the healthy brain and has been considered as a disease biomarker. The strong carotenoid peaks at 1159 and 1517 cm^−1^ decreased or shifted in the chemical vibrational bands in glioma tissue. These changes were used to distinguish glioma and tumor boundaries in our previous study. In this study, Raman peaks of carotenoids and the differences among different types of tissues were also observed, which shows that the new system is promising for intraoperative measurement. However, due to the complexity of tissue properties and the challenge in tissue classification, using a single Raman peak or using peaks due to a single molecular component may not lead to optimal classification. Therefore, we evaluated the classification performance using different combinations of multiple features based on Raman peaks and PC spectra, respectively. It was shown that combinations of Raman peaks due to different biochemical components including a peak of carotenoids led to optimal results. In fact, cancer diagnosis based on the combination of multiple features is a common practice in pathological analysis.

The lipid concentration decreases and its composition also changes during transformation from normal brain tissue into different glioma types. Early reports on brain tumors have highlighted the perturbations in lipid metabolism and a decreased level of lipids that are associated with tumor malignancy. As found in our report, the ratio of phosphorylated protein to fatty acid (I_1584_/I_1442_) increased with the tumor grade progression. The ratio of RR peaks of protein (and collagen) to fatty acid (I_2934_/I_2885_) also increased with the increase in glioma grade. These RR characteristics reveal the importance of the changes in the lipid and protein metabolism in the tumor cells and tissues, which is consistent with our previous reports [26,45,46,47].

In summary, this study has demonstrated the potential of the portable VRR-LRR^TM^ Raman analyzer in intraoperative glioma diagnoses. This technique does not require tissue processing or labeling before spectral acquisition, thus providing a fast and low-cost alternative diagnosis method. It can provide the neurosurgeon with fast and reliable feedback. The intraoperative detection of cancerous tissue would be of great benefit to supplement intraoperative decision making and pathological evaluation.

In this study, surgical margins were defined based on the surgeon’s experience, visual observation, and neuro-navigation. This is a common practice for glioma surgery [82]. The negative control tissues were collected from surgical margins. Pathological analysis was performed to confirm that these tissues were healthy tissues. We observed that Raman peaks due to carotenoids, proteins, and lipids from the margin tissues were different from those in the tumor tissues. Similar Raman spectral changes were also observed in skin, breast, and heart tissue in our previous studies [26,34,40,41,45,46,47].

Despite being one of our largest cohorts of patients with glioma subjected to Raman analysis to date, the small case number is still a limitation. A larger study needs to be undertaken to improve the diagnostic accuracy, efficiency, and feasibility of the intraoperative technique, and to study the sub-types of glioma. Secondly, although spectra were acquired from sites of specimens avoiding blood, blood contamination in the spectra is still inevitable. Further study is needed to explore the effect of blood in VRR in different grades of glioma. Thirdly, the 2021 WHO classification classified adult glioma into astrocytoma (IDH-mutant), oligodendroglioma (IDH-mutant, and 1p/19q-codeleted), and GBM (IDH-wildtype). We will report our recent study on the diagnosis of glioma with different IDH status in another paper.

## 6. Conclusions

In conclusion, we developed a low-cost VRR-LRR^TM^ Raman analyzer (Model# LRR2000) with a handheld optical-fiber probe based on VRR spectroscopy, which can potentially be used for rapid or real-time label-free in situ brain glioma detection. We conducted the first pioneering intraoperative study using the new VRR Raman system and human brain tissues immediately after removal in surgeries. Raman peaks due to key biomolecules were observed and were consistent with our previous ex vivo human glioma studies using commercial confocal micro Raman imaging systems. We have demonstrated that the VRR-LRR^TM^ analyzer has the potential to classify gliomas at different grades from normal brain tissues and may be used to differentiate glioma boundaries. The accuracy is moderate at the current stage, and may be improved in future studies by re-designing the system to improve the signal level.

Using VRR, we evaluated the biochemical composition and changes in the concentrations of biomarkers during glioma’s metabolic process, which may be used to create criteria for margin detection and grading for glioma. By investigating the VRR enhancement in the new molecular biomarkers of 3174 and 3224 cm^−1^, we found that the RR enhancement on the two structures of the FR doublet may be due to oxygen- and hydrogen-bonding effects. An important new biomarker for glioma was found at 3058 cm^−1^ due to a resonance Raman mode of tryptophan.

Although some of the proposed biomarkers observed in this study were consistent with those in our previous ex vivo studies using commercial confocal micro-Raman imaging systems, considering the more complicated environment of the tissue in the intraoperative condition, such as the presence of blood, and this study being the first evaluation of the home-built VRR system, as well as the limited sample size in this study, the findings from this study and the efficacy of the intraoperative diagnosis method using the home-built system should be further verified in future studies.

Nevertheless, this study shows that the proposed VRR-LRR^TM^ Raman analyzer has the potential to be employed as a routine detection approach with a wide range of applications, such as a new optical molecular pathology assay, or an ideal tool for rapid intraoperative in situ glioma diagnosis.

LRR2000 is a new portable analyzer based on VRR with a handheld optical-fiber probe that can be implemented in the operating theatre. We can detect tissue spectra in the operating room immediately after tumor removal, without processing the specimen. Intraoperative information about the glioma grading, sub-typing, and tumor boundary is essential for neurosurgeons, since maximal safe tumor resection and the glioma grade are two important factors that affect tumor progression. VRR provides a potential alternative method for intraoperative diagnosis that would aid neurosurgeons to tailor their surgical strategies to each patient’s tumor profile.

## Figures and Tables

**Table 1:** Number of samples and spectra used in the classification analysis.

Tissue Type	No. of Samples	No. of Spectra
Normal	7	26
Grade 2	6	39
Grade 3	15	92
Grade 4	31	202
Total	59	359

**Table 2:** Peak-SVM and PCA-SVM classification results.

Binary Classes		N vs. C	N vs. G2	N vs. G3	N vs. G4
Peak-SVM	Peaks (cm^−1^)	1371, 1512, 3002, 3474	1620, 3474, 3882	1302, 1371, 3210	1302, 1371, 1512, 3002
	Sensitivity (%)	99.1	94.9	92.4	98.5
	Specificity (%)	50.0	80.8	65.4	57.7
	Accuracy (%)	95.5	89.2	86.4	93.9
	AUROC (%)	75.5	92.4	72.7	77.3
PCA-SVM	PCs	1, 9, 19	3, 10, 13	1, 5, 9, 24	1, 2, 8
	Sensitivity (%)	96.9	94.9	90.2	97.0
	Specificity (%)	50.0	76.9	61.5	53.8
	Accuracy (%)	93.1	87.7	83.9	92.1
	AUROC (%)	74.7	94.6	81.6	80.9
**Multiclass**		**N vs. G2 vs. G3 vs. G4**			
Model		Peak-SVM		PCA-SVM	
Peaks (cm^−1^)/PCs		1512, 3210, 3497, 3541, 3847		1, 4, 10, 14	
Accuracy	N (%)	50.0		42.3	
	G2 (%)	56.4		43.6	
	G3 (%)	37.0		40.2	
	G4 (%)	73.8		90.1	
	Total (%)	60.7		68.8	

N: normal; C: cancer; G2, G3, G4: grade 2, 3, 4.

## Data Availability

The data presented in this study are available on request from the corresponding author(s).
